# Very Late Relapse of Testicular Tumour in Combination with Renal Cancer and Their Retroperitoneoscopic Removal

**DOI:** 10.1155/2011/164070

**Published:** 2011-09-29

**Authors:** Mihály Murányi, Morshed Ali Salah, Béla Tállai, Mátyás Benyó, Tibor Flaskó

**Affiliations:** Department of Urology, University of Debrecen Medical School and Health Science Center, Nagyerdei krt. 98, Debrecen 4032, Hungary

## Abstract

Late relapse of a testicular cancer is an uncommon occurrence. We report a case of late relapse of a testicular tumour combined with a renal cancer and their successful removal with retroperitoneoscopy. The 36-year-old patient underwent left orchiectomy, retroperitoneal lymph node dissection, and chemotherapy, because of mixed tumor including teratoma and embryonal carcinoma. 18 years after the successful primary therapy elevated serum alpha-fetoprotein level had been confirmed, then MRI and PET-CT scans demonstrated a 30 mm left renal mass and 22 mm retroperitoneal lymph node above the bifurcation of the left common iliac artery. We performed retroperitoneoscopic lymph node dissection and left renal tumour resection in the same session. The histology revealed embryonal carcinoma for the retroperitoneal lymph node and renal cell carcinoma for the left renal mass. We can conclude that late followup of patients with testicular tumour is important. Retroperitoneoscopy is feasible approach for the removal of retroperitoneal lymph node metastasis and resection of renal tumor.

## 1. Introduction

Most recurrences of testicular cancer after curative therapy occur in the first 2 years [1]. Recurrences are uncommon after 2 years. Late relapse is defined as tumour recurrence more than 2 years after complete remission following primary treatment. Many years after successful treatment, second primary malignancies can occur [2–4].

Herein we report a case of late relapse of a nonseminomatous germ cell tumour 18 years after first complete remission, combined with a renal cancer and their successful treatment with retroperitoneoscopy.

## 2. Case Report

The 36-year-old patient had a history of left radical inguinal orchiectomy, open extended bilateral retroperitoneal lymph node dissection, and chemotherapy because of testicular cancer before 18 years in another center. The histology revealed a mixed tumor including teratoma and embryonal carcinoma in the left testicle and in the paraaortic lymph nodes. The treatment was successful. During 12 year follow-up period, there was no recurrence. 

**Figure 1:**
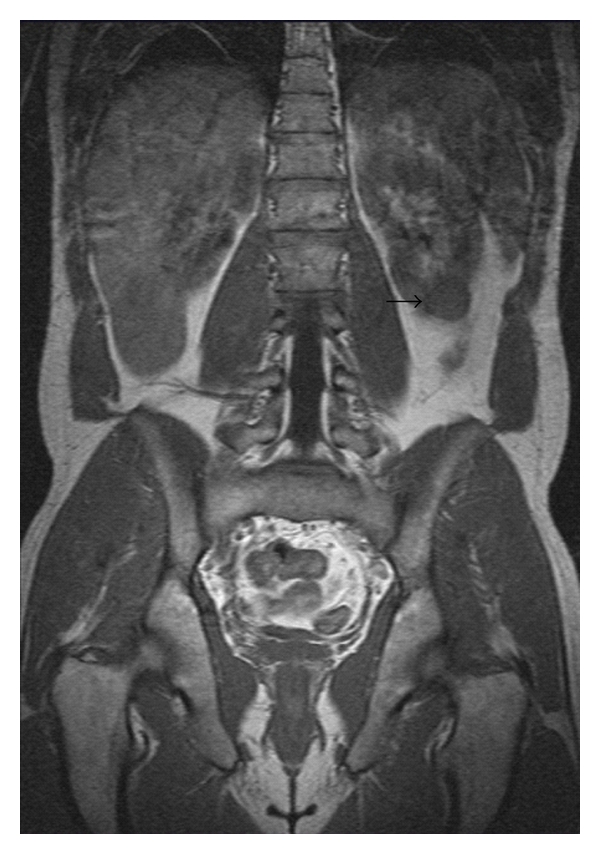
Coronal magnetic resonance imaging scan demonstrating 30 mm left renal mass (arrow).

**Figure 2:**
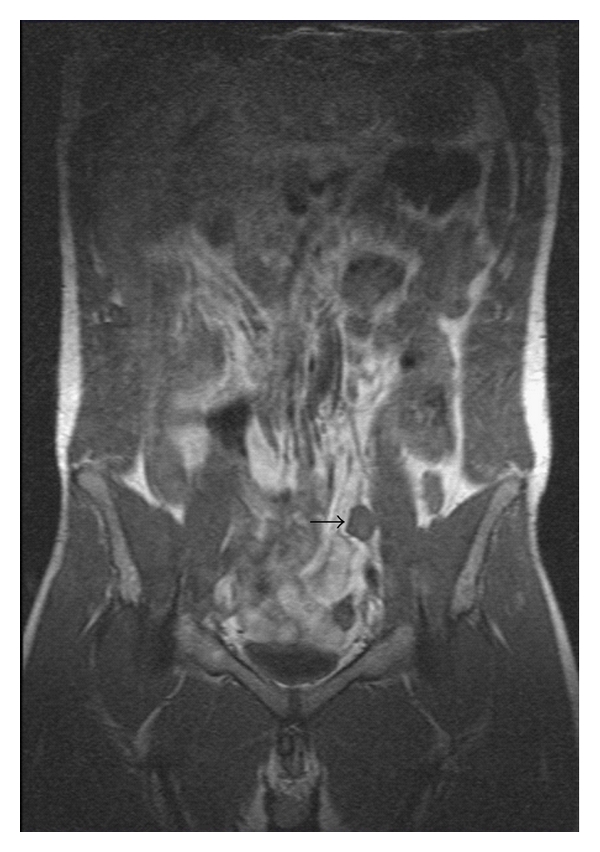
Coronal magnetic resonance imaging scan demonstrating 22 mm retroperitoneal lymph node (arrow).

18 years after the primary therapy, elevated serum alpha-fetoprotein level (50, 24 ng/mL) was confirmed. By physical examination, there was no abnormality. MRI scan demonstrated a 30 mm left renal mass (Figure 1) and 22 mm retroperitoneal lymph node above the bifurcation of the left common iliac artery (Figure 2). PET-CT scan confirmed this finding and showed intensive fluorodeoxyglucose uptake in both lesions. At that time, he was referred to our center. We performed retroperitoneoscopic lymph node dissection and left renal tumor resection at the same session. The operation time was 285 minutes, and blood loss was 150 mL. Complication did not occur. Transfusion or conversion to open surgery was not necessary. The postoperative period was uneventful, analgesic requirements were moderate, and hospital stay was 3 days. The histology revealed embryonal carcinoma for the retroperitoneal lymph node and renal cell carcinoma for the left renal mass. The renal cell carcinoma was independent of the germ cell tumour. Immunohistochemical staining showed that tumour cells from the retroperitoneal lymph node were negative for vimentin, CD10, and CD30, in contrast with tumour cells from the renal mass, which were positive for vimentin and CD10 and negative for CD30. Six months after the operation, the patient had no recurrence and serum alpha-fetoprotein level was normal.

## 3. Discussion

Late relapses of nonseminomatous germ cell tumours are rare 1–6% of patients have a late relapse [5–8]. Late relapse may occur at any time, even 32 years after primary treatment [6, 9].

The most frequent location of late relapse is retroperitoneal space. The lungs and the mediastinal lymph nodes are rarely affected [1, 6, 8, 10]. After retroperitoneal lymphadenectomy, relapse in the retroperitoneum is rare, the most likely site of recurrence being the chest [11].

The optimum treatment for the patient depends on the primary treatment, histological type of the tumour, and the location of late relapse. The surgical approach seems to be the best choice. Survival of patients with late relapse is better, if they undergo complete surgical resection [1, 6, 10, 12, 13].

Both seminomatous and nonseminomatous germ cell tumours can relapse after 2 years. Late relapse with nonseminomatous germ cell tumour patients is more frequent [7, 10]. Teratoma is the most common type of neoplasm in late recurrences, followed by yolk sac tumour. Other types of testicular tumours, including embryonal carcinoma, seminoma, choriocarcinoma, and nongerm cell malignant tumours occur rarely [14].

After the introduction of cisplatin-based chemotherapy for the treatment of testicular germ cell tumours, the survival of patients increase dramatically. Recently, the cure rate is excellent, even in the advanced stage. Testicular germ cell cancer occurs predominantly in young adulthood; therefore, these patients may experience high levels of risk as they reach older ages [15]. It resulted in increased focus on the serious treatment-related long term side effects, which can cause higher mortality rates. Many years after successful treatment, second primary malignancies can occur as the most serious late treatment side effect. Testicular cancer survivors are in greater risk of developing secondary tumours compared to the general population [2–4]. An increased incidence has been found following both radiotherapy and chemotherapy [2].

## 4. Conclusion

We can conclude that late followup of patients with testicular cancer is important. Patients treated for testicular cancer may need annual follow-up evaluations throughout their life because of the possibility of late relapse and second primary malignances as well. Retroperitoneoscopy is feasible approach for the removal of retroperitoneal lymph node metastasis and renal tumor resection even after open transperitoneal lymph node dissection.
